# Acute and chronic rhinosinusitis and allergic rhinitis in relation to environment, comorbidity and ethnicity

**DOI:** 10.1186/2045-7022-5-S4-P26

**Published:** 2015-06-26

**Authors:** Ruth Hoffmans, Deniz Hastan, Kees van Drunen, Wytske Fokkens

**Affiliations:** 1 Academic Medical Centre, otorhinolaryngology, Amsterdam, Netherlands

## Aims

This study was conducted to assess the relation between allergic rhinitis (AR), acute rhinosinusitis (ARS) and chronic rhinosinusitis (CRS) and environment, comorbidity and ethnicity.

## Methods

A posted GA2LEN screening questionnaire was sent to all those in a random sample of Dutch population (n=16700) in three different areas.

## Results

The prevalence of ARS is significantly related to AR, a doctor’s diagnosis of CRS, urticaria, eczema, smoking, gender, ethnicity and age. The prevalence of CRS is significantly related to AR, a doctor’s diagnosis of CRS, urticaria, adverse response to painkiller, smoking, ethnicity, asthma and age. The prevalence of AR is significantly related to a doctor’s diagnosis of CRS, urticaria, eczema, adverse response to painkillers, smoking, occupation, ethnicity, asthma, age, CRS and ARS

## Conclusion

Some environmental factors, comorbidity and ethnicity are positively or negatively related to AR, ARS and CRS. Place of residence in the Netherlands is not related to the prevalence of these diseases.

